# Infants Time Their Smiles to Make Their Moms Smile

**DOI:** 10.1371/journal.pone.0136492

**Published:** 2015-09-23

**Authors:** Paul Ruvolo, Daniel Messinger, Javier Movellan

**Affiliations:** 1 Olin College of Engineering, Department of Engineering, Needham, Massachusetts, United States of America; 2 University of Miami, Department of Psychology, Coral Gables, Florida, United States of America; 3 Institute for Neural Computation, University of California San Diego, La Jolla, California, United States of America; University of Portsmouth, UNITED KINGDOM

## Abstract

One of the earliest forms of interaction between mothers and infants is smiling games. While the temporal dynamics of these games have been extensively studied, they are still not well understood. Why do mothers and infants time their smiles the way they do? To answer this question we applied methods from control theory, an approach frequently used in robotics, to analyze and synthesize goal-oriented behavior. The results of our analysis show that by the time infants reach 4 months of age both mothers and infants time their smiles in a purposeful, goal-oriented manner. In our study, mothers consistently attempted to maximize the time spent in mutual smiling, while infants tried to maximize mother-only smile time. To validate this finding, we ported the smile timing strategy used by infants to a sophisticated child-like robot that automatically perceived and produced smiles while interacting with adults. As predicted, this strategy proved successful at maximizing adult-only smile time. The results indicate that by 4 months of age infants interact with their mothers in a goal-oriented manner, utilizing a sophisticated understanding of timing in social interactions. Our work suggests that control theory is a promising technique for both analyzing complex interactive behavior and providing new insights into the development of social communication.

## Introduction: Mother-Infant Interaction Study

Interactive smile games between infants and their caregivers mark an important milestone in infant social development and serve as the foundation for later forms of social interaction [1, 2]. Research on mother-infant smile interaction has used statistical methods to describe infant-mother responsivity and turn-taking [3–5]. While providing important insights into interactive structure, it remains unclear why mothers and their infants time their smiles as they do. Here we quantify in an objective manner whether the smiles of young infants are purposeful and attempt to discover what their purpose might be. Detecting goals in observed behaviors may provide a deeper understanding of typical social development, and of early-emerging disorders such as autism.

The rich literature on smiling in face-to-face interactions between parents and infants under seven months of age is predominantly descriptive [6]. Duration-focused research has documented the tendency of infant and mother smiling to co-occur, but says less about which partner is responsible for the occurrence of joint smiling [7, 8]. Research focusing on the frequency of smile onsets indicates that mothers tend to respond to infant smile onsets with a smile of their own [1, 9, 10]. Infants have a similar though weaker tendency to smile in response to mother smiles. It is unclear, however, how long infants maintain these responsive smiles. The current research offers a comprehensive examination of smiling patterns which integrates the duration and frequency approaches. Smile onsets (and offsets) are investigated with respect to their role in influencing the likelihood of subsequent smiling states characterized by the durations of their co-occurrences.

How should the smiling goals of young infants be investigated? Interactive goals are thought to emerge with the development of intentional communication [11–13]. Typically, the concerted use of gestures to influence another’s actions—e.g., requesting a toy—constitutes the first evidence of intentional communication [11]. This type of intentional gesturing emerges between 8 and 12 months of age. During this period, infants also begin to show anticipatory smiling in which they gaze at an object, smile, and then turning the already smiling face to look at the mother, which is associated with means–ends gesturing to others [14–16]. However, infants four months of age and under do not use gestures in a concerted fashion to achieve goals, nor do they engage in anticipatory smiling. They do, however, engage in a rich interplay of smiling behavior with their parents. Are these smiles timed in a fashion that tends to produce particular dyadic end states?

In the mid 20^th^ century Norbert Weiner pioneered the development of Cybernetics, whose focus was the mathematical study of purposeful, goal-oriented behavior in animals and machines [17]. His pioneering work was influential in the development of Control Theory, the formal machinery behind modern robotics and automation [18–21]. A simple case of a controller is a thermostat. Its input is the room temperature and its output is a command for the heating system to turn on or off. The goal (or purpose) of the controller is to keep the room temperature within a desired range. In the current context, goal and purpose refer to hypothesized end states. The question at hand is whether the controller (e.g., the infant or mother) times its actions in a fashion consistent with producing those states. The use of the terms goal and purpose does not imply that the infant or mother are aware of these states.

Advances in machine learning and more powerful computers have recently been used to reverse engineer the goals of controllers from their behavior. The use of control theory to reverse-engineer the goals of an agent from observations of its behavior is known as “inverse optimal control” [18–20]. The inputs to inverse optimal control algorithms are time-stamped sequences of sensory data and observed behaviors (e.g., the temperature of the room and the commands sent by the thermostat). These inputs are used to quantify whether there is evidence that the observed behaviors are goal-oriented and, if so, to infer what the goals are.

In inverse optimal control, we typically use the term “agent” to refer to the system whose goals we want to discover, and “plant” to refer to the system that the agent is trying to control. While inverse optimal control algorithms are mathematically and computationally complex, their logic is simple. First, we develop a dynamical model of the plant [22]. The result is a model that predicts how the plant behaves under different conditions. Second, we formulate a *hypothetical goal* for the agent whose behavior we are trying to understand. Third, we use control theory [23] to find the optimal control policy to achieve the *hypothesized goal*. Fourth, we determine how closely the agent’s actions match the actions dictated by the optimal policy. We iterate steps two through four over a space of potential goals. If the optimal policy for a particular goal matches the observed actions better than the optimal policies corresponding to the other potential goals, then we conclude that the agent’s actions are directed towards that particular goal. Conversely, if the actions equally match all possible goals, there would be no evidence for goal-directedness on the part of the agent.

Here we apply inverse optimal control methods to discover whether infants and mothers time their smiles in a purposeful manner and if so, what their purpose might be. To this end, we model the smile interactions between mothers and infants as a game between two agents where each agent is trying to control a plant (the other agent) to achieve its goals. When analyzing the purpose of the mother’s behavior we treat her as the agent and the infant as the “plant” she is trying to control. When analyzing the purpose of the infant’s behavior we treat the infant as the agent and mother as the “plant” that the infant is trying to control. The four goal states we compared (mother smiling, infant not smiling; infant smiling, mother not smiling; both smiling; neither smiling) represent a logical division of possible dyadic smiling states. To ensure that infants could see mother’s smiles we constrained our analysis to interactive periods in which infants were gazing at mother.

## Methods: Mother-Infant Interaction Study

Thirteen infant-mother dyads were observed in weekly face-to-face interactions between the ages of 4 and 17 weeks [8]. Mothers provided written informed consent, and the Purdue University Institutional Review Board approved all procedures. For each of the 13 dyads we computed the probability of 4 hypothetical goals separately for each agent (mother and infant): (1) maximize the time of mother smiling / infant smiling (simultaneous smiling), (2) maximize the time of mother smiling / infant not smiling, (3) maximize the time of mother not smiling / infant smiling, and (4) maximize the time of mother not smiling / infant not smiling. The basic logic of how we computed these probabilities follows the four steps described in the introduction. For instance, in the case of determining the probability of each goal for the infant, we perform the following steps: (1) fit a predictive model of mother’s smiling behavior, (2) hypothesize a particular goal for the infant, (3) compute the optimal smile timing for the infant to realize that goal as well as possible, and (4) determine how well the optimal smile timing fits the empirically observed smile timing. For complete details see *S*1 *Methods*. Probabilities were compared with repeated-measures analysis of variance (using Greenhouse-Geisser degree of freedom corrections when appropriate), and followed-up with two-tailed *t*-tests; *p*-values below .05 were considered significant. Significant differences between values indexing the probability of each of the 4 goals would provide evidence that the observed behaviors served to achieve that goal.

## Results: Mother-Infant Interaction Study

**Fig 1:**
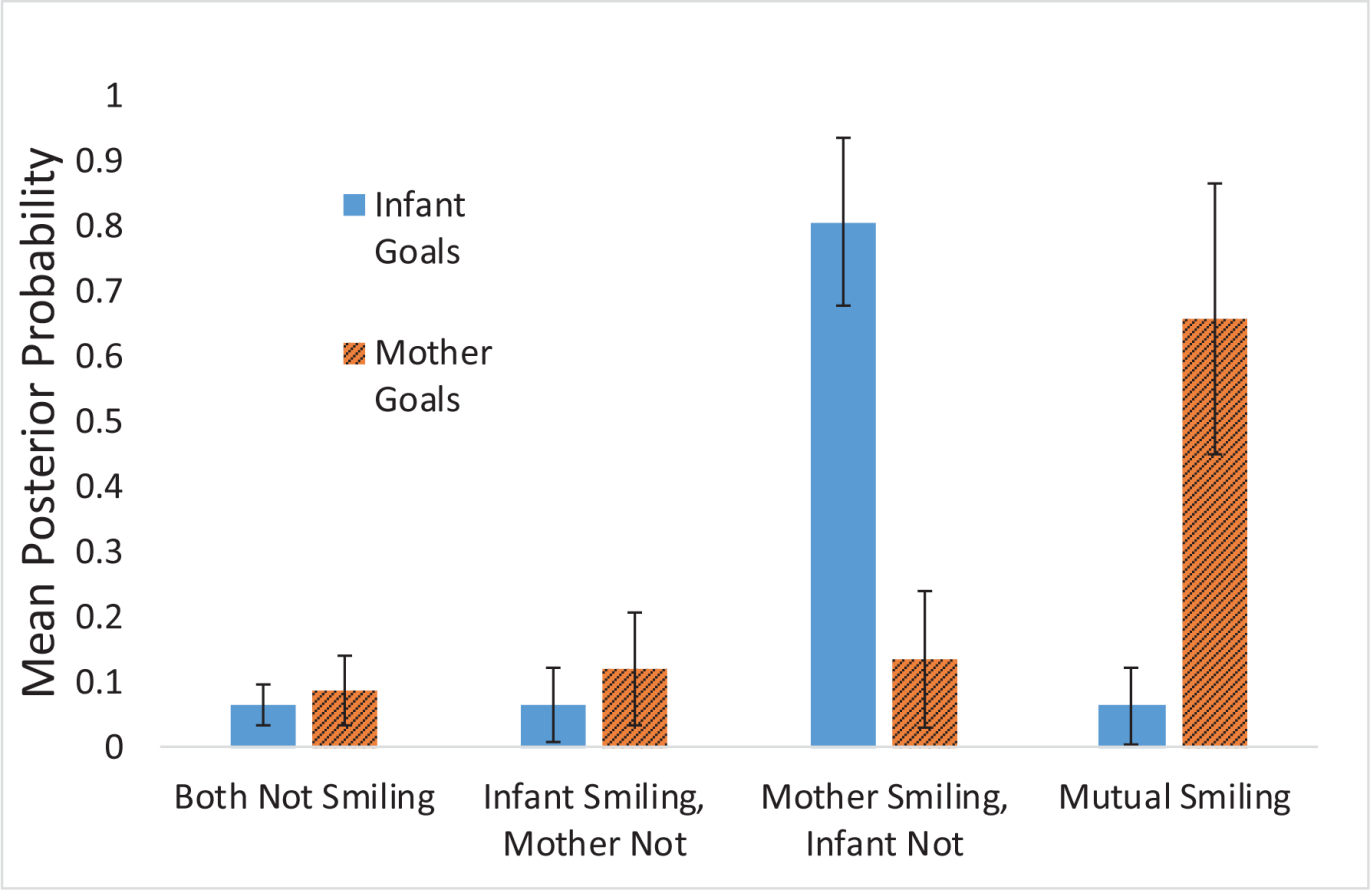
Comparison of Infant and Mother Goals. Means of the probability distributions of potential mother and infant goals. Error bars are 95% confidence intervals of the mean.

An ANOVA of the probabilities produced by the inverse optimal control algorithm for each of the four goals revealed a significant effect of goal for both mothers (*F*(1.13,13.55) = 13.79, *p* = .002, η^2^ = .54) and infants (*F*(1.08,12.99) = 66.96, *p* < .001, η^2^ = .85). For mothers the goal of maximizing simultaneous smiling had significantly higher probabilities (see **Fig 1**) than each of the three other goals (t(12) = 4.77, p < .001 in comparison to maximizing simultaneous non-smiling, t(12) = 3.38, p = .005 in comparison to maximizing infant-only smiling, and t(12) = 3.70, p < .001 in comparison to maximizing mother-only smiling). Further, for 10 out of the 13 mothers maximizing simultaneous smiling was their most probable goal (see **Table 1**). This prevalence significantly differed from the chance level of 0.25 (binomial test p = .00013).

**Table 1:** Inferred goal for mothers and infants. Shown are the proportion of mothers and infants who, according to our Bayesian model, exhibit behavior consistent with a goal of maximizing time spent in a particular smile configuration.

	Both Not Smiling	Infant-only Smiling	Mother-only Smiling	Mutual Smiling
Mothers	0/13	1/13	2/13	10/13
Infants	1/13	0/13	11/13	1/13

The same analysis applied to infants revealed that for infants only the goal of maximizing mother-only smiling had significantly higher probabilities (see **Fig 1**) than each of the three other goals (t(12) = 9.93, p < .001 in comparison to maximizing simultaneously non-smiling, t(12) = 7.92, p < .001 in comparison to maximizing infant-only smiling, and t(12) = 6.93, p < .001 in comparison to maximizing simultaneous smiling). Further, for 11 out of the 13 infants maximizing mother-only smiling was the most probable goal (see **Table 1**). This prevalence significantly differed from the chance level of 0.25 (binomial test p < .001). That is, infants had the goal of creating and maintaining states in which they were being smiled at (by their mothers) but were not smiling themselves (see **Fig 1**).

**Fig 2:**
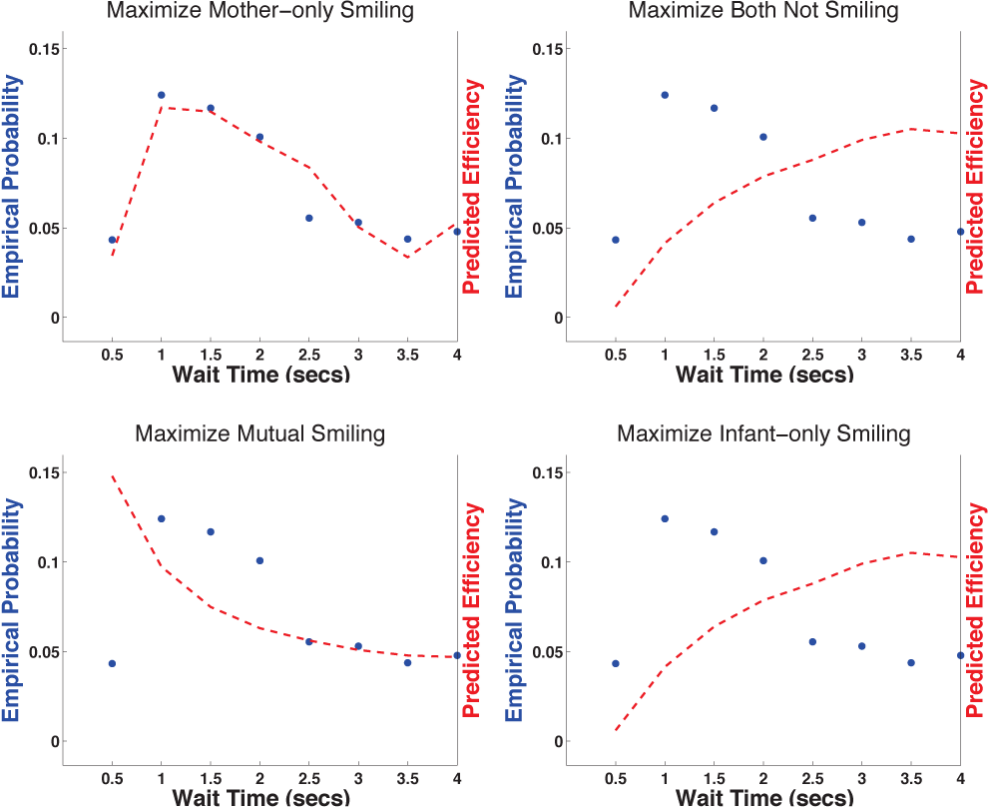
When to Smile? Performance for different strategies derived from different possible infant goals (each displayed in a different panel) versus the observed probability of infant actions. Mother is smiling and infant has just stopped smiling. On the x-axis are wait times until the infant smiles again. The y-axis displays the modeled performance (dashed lines) of various wait times (different plots show different possible goals) versus the empirical probability (dots) that the infant selects a particular wait time.

Infants exhibited sophisticated timing behaviors to achieve their goals. For example, consider the wait time distributions displayed by infants initiating a smile given that mother was smiling (**Fig 2)**. Fig 2 displays the empirical distribution of infant wait times in this context and the efficiency of each of these wait times in achieving each of the four goals analyzed. Infant smile initiations peaked at 1 second and then decayed. The posterior probability values indicated that infants prefer the configuration in which mother is smiling and infant is not. Consequently, one might expect that infants would be unlikely to ever smile when mother is already smiling. However, this was not the case. Implementing maximally efficient infant wait times before smiling involves optimal tradeoffs between immediate and long-term goal maximization. An infant strategy of never smiling maximizes immediate time in the mother-only smiling configuration, but at the potential expense of future time in the mother-only smiling configuration (because mother is likely to cease smiling after a number of seconds of smiling alone). Predictions derived from the goal of maximizing “mother-only” smiling appeared to maximize immediate seconds of mother-only smiling while minimizing the probability of mother terminating her smile.

## Introduction: Human-Robot Interaction Study

**Fig 3:**
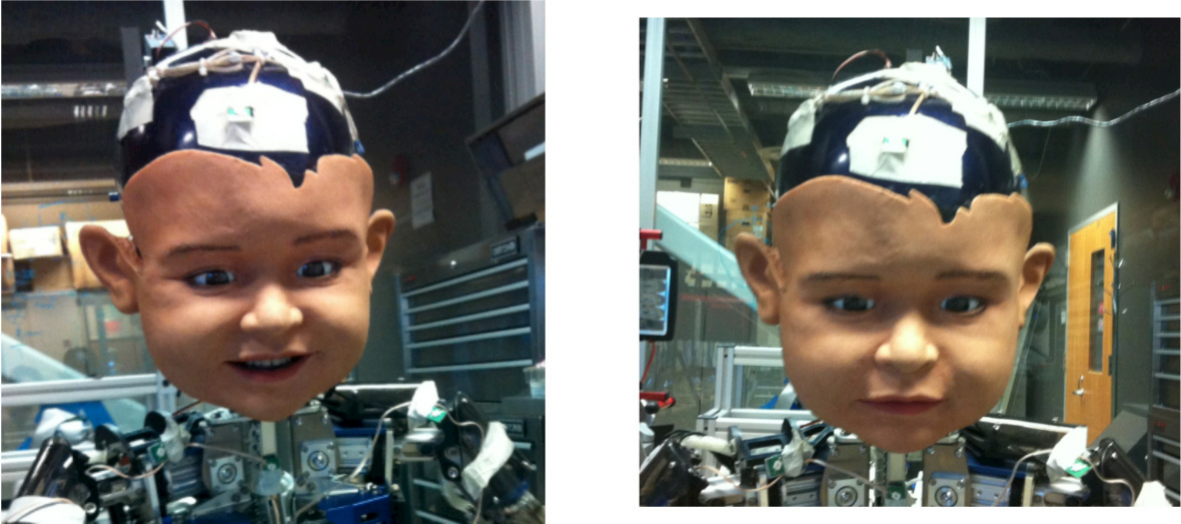
Diego-San’s Expressions. Diego-San, the robot used to interact with adults smiling (left) and not smiling (right).

To further validate the results obtained using the inverse optimal control methods, we programmed a sophisticated child-like robot, named Diego-San, to play smile games with adults (see **Fig 3**). The key control program implemented the infant control strategy inferred from infant-mother interaction. This strategy was expected to maximize adult-only smiling, the corollary of mother-only smiling from the mother-infant study. Alternates to the infant control program were replay, mirror, and infant plus. Replay was a null hypothesis non-contingent model that enacted behaviors previously displayed to a previous participant. The mirror program enacted perfect smiling responsivity, smiling and not smiling to match the undergraduate’s smiling state. Infant plus was a hybrid program based on the infant model with increased likelihood of mirroring smiling and non-smiling states, and was designed to provide a powerful alternative to the infant program.

## Methods: Human-Robot Interaction Study

UC San Diego undergraduates (N = 32) participated in the study. Each participant gave written consent, and UC San Diego’s Institutional Review Board approved the experiment. Each participant interacted with the robot for four counterbalanced periods. Each period lasted 3 minutes during which the robot tracked the participant's face using a combination of saccadic eye movements and head movements. In each period, the robot smiled according to one of the four control strategies detailed below:

*1- Infant*: The robot smiled using the control policy synthesized from the inverse optimal control analysis of infant smile behavior described in the previous section.
*2- Replay*: The robot smiles were locked to the timing of the smiles generated during the session with the *previous* participant using the Infant controller. Notably, there was no contingency between the participant's smiling and Diego-San’s smiling.
*3- Mirror*: In this condition Diego-San always matched his smile to that of the participant, as if he were a “smile mirror”.
*4 Infant Plus*: The smile timing of this controller was identical to *Infant* with the modification that Diego-San was more likely to modulate his expression to be the same as the participant (elevated probability of matching of 50% per second). This controller was designed to test the effect on the participants of increasing the contingency of the robot’s smiling to the participants’ smiling (as compared to the Infant controller).

The robot’s perception of both facial location and facial expression (i.e. smile versus not smile) was given by the output of an automated facial expression recognition system, the Computer Expression Recognition Toolbox (CERT) [24]. CERT output was also used to quantify human smiling while interacting with the robot during each 3-minute period. After each period of interaction, we also administered a questionnaire to probe the participants' experiences interacting with Diego-San. The questionnaire [25] consisted of twenty-one five-point Likert scale items (e.g. is the robot *1—apathetic* or *5—responsive*) that we summed to give an overall rating of positivity of a particular participant toward Diego-San implementing a particular control strategy.

## Results: Human-Robot Interaction Study

First we assessed how the time spent in each of the four smile configurations (robot and human smile, robot smiles human does not smile, robot does not smile, human smiles, neither robot nor human smile) correlated with the participants’ positive rating of the interaction with the robot. The only significant correlation was between amount of time spent in simultaneous smile and positivity rating (*r* = .57,*p* < .001). Thus, the human participants appeared to have similar preferences to the ones we had previously found in mothers: they rated their experience with the robot more positively when the robot simultaneously smiled with them.

Next, we investigated the effect of the 4 controllers on the amount of time that the robot-human pair spent in the adult-only smiling state. We found a significant main effect of the controller on the duration of participant-only smiling (F(3,93) = 10.20, p < .001, η^2^ = .25). Subsequent paired t-tests revealed that the duration of participant-only smiling was significantly longer for the controller based on the inferred infant goals than for each of the other 3 control conditions (t(31) = 2.72, p = .011 in comparison to replay, t(31) = 4.78, p < .0014 in comparison to mirror, and t(31) = 3.14, p = .004 in comparison to infant plus). Thus a controller embodying infant goals derived from mother-infant interaction had the predicted effect when transplanted into a robot that interacted with adult humans: it maximized the amount of adult-only smiling just as infants had maximized mother-only smiling.

## Discussion

The current study provides an innovative and rigorous mathematical analysis of smiling patterns in infants four months of age and younger. Control theory methods were used to objectively ascertain whether observed behaviors were purposeful and to infer their specific purpose. Mothers timed their smiles to maximize periods of simultaneous smiling with their infants. By contrast, infants timed their smiles to maximize periods of mother-only smiling.

We found that infant (and mother) timed their smiles in a sophisticated manner that demonstrated mastery of the statistics of social interaction. Both partners timed their smiling to have a systematic impact on the other partner. We do not claim, however, that either partner was aware of timing their smiles in order to achieve a specific goal. Although Piaget [26] was explicitly interested in the development of mental representations of goals, modern behavioral researchers are agnostic as to whether the infant is or is not aware of the end state she or he is pursuing [27]. These results nevertheless suggest that infants leveraged social interaction patterns to achieve specific dyadic states well before there is evidence for conventional use of means-to-ends behavior.

The four goal states we compared (mother smiling, infant not smiling; infant smiling, mother not smiling; both smiling; neither smiling) represent a logical division of possible dyadic smiling states. However, other goals are possible. Infants may have dynamic goals such as changing mother’s smiling state (whether or not she is smiling). Infant goals may also be unmeasured correlates of the goals we examined (such as producing a particular type of mother smile). Nevertheless, amongst the four compared goals there was clear evidence that some goals were more consonant with the data than others. To further validate our analysis, we ported the smile timing strategy used by infants into a robot that engaged in smile games with adult humans. The transplanted smile timing strategy replicated the infant-mother study: it maximized adult-only smiling.

Mothers’ goals of maximizing simultaneous smiling suggest that mutual states of joint positive engagement are highly valued. These mutual states are likely one of the motivations for mothers’ high levels of smiling responses to infant smile onsets. The infant goal of maximizing mother-only smiling may be construed in behavioral ecological terms [28]. The infant smile may signal a desire for affiliative signals from the mother such as smiling. When mother smiling occurs, the infant smiling signal is no longer required. It is also possible that infants tend to cease smiling after a mother smile in order to regulate positive affective arousal [29].

Mothers' and infants' goals differed. Mothers acted consonant with the goal of increasing mutual smiling while infants acted consonant with the goal of increasing mother-only smiling. Interaction involves emergent patterns that may differ from those favored by either party. Common dyadic patterns in infant-mother interaction may, in part, results from the juxtaposition of these different goals. The goal state that had similar posterior probability values for infant and mother was that in which neither infant or mother were smiling, a statistically common state of affairs in infant-mother interaction [1]. The conflict between mother and infant goals is relevant to a long-standing debate concerning infant responsivity. Early theorists were concerned that although infants appeared to alter their behavior in response to their parents, parents tended to time their actions in a fashion that made infant appear to be responsive [30]. Here we show that infants act in pursuit of goals that differ from parent goals, highlighting the role of the infant as an independent interacting agent.

The study of goals during interaction typically begins with the emergence of intentional gesturing. Gestures are said to be intentional when they are used as means to an end. Infants might, for example, extend their arms to request a toy or snack. Such behavior emerges between 8 and 12 months in typically developing infants [14–16]. A similar difficulty exists in understanding manual gesturing to obtain food items observed among orangutans [27, 31] and chimpanzees [32]. The non-human primate literature focuses on the specificity of signal-response associations between two individuals [33]. Here we build on the logic of signal-response associations using an inverse optimal control framework. This framework provides a quantitative foundation for pairing signaling actions and responses.

The methods employed here provide support to the idea that by 4 months of age infants smile in a purposive fashion, and clarifies what those purposes are. The discovery of goal-oriented interactive behavior in infants under six months of age—and the elucidation of the goals underlying those behaviors—could shed light on our understanding of the development of typical and atypical social behavior [34]. For instance, the approach presented here could be used to analyze the interactive behavior of children who are at high risk for developing autism spectrum disorders (ASD) [35]. The analysis could help disambiguate whether young infants who will go on to develop an ASD have more object-oriented and less socially-oriented goals than infants who do not go on to an ASD outcome [36].

## Supporting Information

S1 Data The supporting data contains the data from both the human robot interaction study, as well as the mother-infant interaction study. (ZIP)

S1 Methods The supporting methods contain a full description of the procedures used to perform the analyses in this paper. (DOCX)
