# Supplementary material for: Infants Time Their Smiles to Make Their Moms Smile
Source: PLoS One. 2015 Sep 23;10(9):e0136492. doi: 10.1371/journal.pone.0136492 (PMC4580458; doi:10.1371/journal.pone.0136492)
Supplement: S1 Methods — (DOCX) [file pone.0136492.s002.docx]

**Supplementary Methods**

**Facial Expression Coding for Mother-Infant Dataset.** The onset and offset of infant and mother smiles were coded continuously for each video frame of observation (30 fps). Coders certified in the anatomically-based Facial Action Coding System reliably coded smiles (Zygomaticus Major contraction) in this extant, longitudinal database [8].

**Inverse Optimal Control Model Details.** In order to apply the inverse optimal control technique of [20], we conceptualized the interaction between mothers and infants as an optimal control with each partner alternatingly assuming the role of the agent or the plant.

*Time:* We adopted a discrete time framework, with time steps separated by 400 milliseconds. There were 12 video frames (one ever 33.333 milliseconds) for each of the fifty 400ms discretized time steps used to create the state space of transition matrices.

*State space:* For each time step, the state consisted of two binary variables and two continuous-valued variables. The binary variables encoded whether or not each partner was smiling at that time step. The continuous-valued variables encode how long infant and how long mother had been in his or her current smiling configuration at that step. A given time step, for example, might involve mother smiling and having been smiling for 5 seconds while the infant is not smiling and has been not smiling for 2 seconds).

We converted the continuous dimensions of the state space by discretizing them into fifty 400ms segments representing the time intervals [0s, 0.4s), [0.4s, 0.8s), [0.8s, 1.2s), … [19.6s, ∞]. To construct the full state space, we take the Cartesian product for each of the individual state components (i.e. the 2 binary smile variables and the 2 temporal variables) yielding a total of 10,000 states (2×2×50×50 = 10,000).

*Plant dynamics:* The plant dynamics indexed by one-step transition probabilities between the different state space values. These probabilities specify the likelihood of each partner initiating or terminating a smile given their own prior actions and those of their partner. We computed the Probabilities using maximum likelihood parameter estimation with temporal pooling. Pooling is required due to data sparsity, which did not allow us to estimate the transition probabilities for each of the 10,000 states independently. Specifically, we fit the transition probabilities independently for each joint smile configuration, but pooled data over similar temporal contexts with that configuration. We estimated the transition over the next 400ms by pooling data over the 16 regions of the two-dimensional space of time since each partner last changed. The particular choice of the regions is motivated by allocating exponentially less temporal resolution as the time since each agent changed smile configurations gets larger. Specifically the set of regions we pooled over is given by the Cartesian product between motherIntervals and infantIntervals where motherIntervals = infantIntervals = {[0, 1.2s], (1.2s, 3.6s), (3.6s, 10.8s], (10.8s, ∞)}. As a result, the transition dynamics models for mother and infant each have 64 model parameters.

*Prior Distribution over Agent Goals:* the prior distribution over goals was modeled as a uniform distribution over a large number of systematically varied utility values assigned to the four possible joint configurations of infant and mother smiles (e.g., both are smiling, mother smiling infant not smiling, infant smiling mother not smiling, both are not smiling). We specify a goal as a vector of 4 utility values (one for each of the 4 possible infant-mother configurations). We used a uniform grid search over 11 possible utility values for each of the 4 configurations yielding a total of 11^4^ considered goals. For example, one goal considered is that an infant derives 1 utility unit when he is smiling with his mother, .5 when he is smiling by himself, .2 when both he and his mother are not smiling, and .8 when his mother is smiling and he is not smiling. In order to calculate the probability that an agent’s goal was maximization of the duration of time spent in some configuration (e.g. simultaneous smiling) we summed the posterior probabilities of all considered goals that had the property that simultaneous smiling had the highest reward value relative to other joint smile configurations (such as both not smiling or mother-only smiling).

*Figure 2.* To quantify the relative efficiencies of various infant smile wait times in Figure 2, we created a simplified model of mother’s behavior in which she had a fixed probability (determined empirically via a maximum likelihood fit to the observed data) of either initiating or terminating a smile per half second of waiting time based in a given mother-infant smile configuration. Likewise, infant smile strategies were parametrized to involve waiting at half second intervals (i.e., .5, 1, 1.5, 2, 2.5, 3, 3.5, or 4 seconds as show on the X axis) and then smiling for two seconds.

**Solution Methods for the Optimal Control Problem.** Given a description of the plant and a hypothesized goal we use dynamic programming [12] to compute the policy, π*, that satisfies:

$$\pi^{*}=\underset{\pi}{\mathrm{argmax}} E\left[ \sum_{t=0}^{\infty} ϒ^{t}R_{t} \right]$$

Where *R*_0:∞_ is an infinite sequence of random variables specifying the value of the performance function at each time step assuming we always choose actions prescribed by the optimal policy and γ ∈ [0,1) is a discount factor specifying the tradeoff between immediate and future.

A by-product of computing the optimal policy is the construction of the optimal state-action value function q. The function q is defined as the expected future reward for executing action a starting in state s and following the optimal policy thereafter:

$$q\left( s,a \right)= r\left( s,a \right)+ E\left[ \sum_{t=0}^{\infty} \Upsilon^{t}R_{t}|S_{0}=s, A_{0}=a,\pi^{*} \right]$$

Intuitively, we can think of the relative values of q(s, a) and q(s,a’) as determining which action, a or a’, is more optimal in the state s for achieving the agent's goal.

*Softmax Action Selection Rule:* Next, we define a function that specifies how likely an agent is to execute a particular action in a particular state given the optimal policy. A simple choice would be to assign probability 1 to the action given the optimal policy and 0 to all others. Here, we assign probabilities to an agent's actions by assuming it employs a softmax action selection rule that allows for some noise in the agent selecting the optimal action at each time step. Specifically, we assume that:

$$p\left( a | s,q \right)= \frac{e^{\tau q\left( s,a \right)+b(a)}}{\sum_{a'} e^{\tau q\left( s,a^{'} \right)+b(a^{'})}}$$

Where τ is a scalar parameter that when close to 0 makes the action probabilities uniform and when close to infinity reverts to predicting that the agent will always choose the optimal action. Additionally, b is a function that specifies a bias for choosing a particular action. The entries of b as well as the value of τ were fit independently for each hypothesized goal to maximize the likelihood of the observed action choices for each agent.

**Human Robot Interaction Experiment Details.**

*Room Setup and Experimental Procedure:* Diego-San was enclosed in a four-sided curtained enclosure to increase the participant's sense of privacy when interacting with the robot. The participant was required to remain seated during the experiment and to remain at least 18 inches from the robot at all times. The participant was seated in a chair with rolling wheels that allowed them to move within the enclosure while remaining seated. The participant interacted with each of the four robot controllers: infant, infant-plus, replay, and mirror. The order of presentation of each controller was counter-balanced to avoid order effects. Following each interaction, a questionnaire was administered (see Dependent Measures).

Diego-San’s eyes were programmed to verge on the face of the participant to give the appearance that the robot is looking at the participant. Diego-San would randomly blink at an average frequency of 0.5Hz.

Upon arriving at the lab, participants were given the following written instructions:

*Researchers at the Machine Perception Laboratory are designing a robot named ``Diego-San''. Diego-San is just beginning to learn how to interact with people. Diego-San has the ability to sense and respond to some of the same social cues that humans use to communicate with each other. Currently, Diego-San can see where people's faces are and whether or not they are smiling. He does not have any other perceptual abilities (such as the ability to detect gestures or sounds). In this experiment you will interact with Diego-San for four 3-minute sessions. During each session, Diego-San will run a different social interaction program. Each program specifies a different pattern for how Diego responds to your actions. Following each interaction, we will administer a questionnaire that asks you to evaluate Diego-San's behavior during the previous 3-minute interaction.*

*Facial Expression Recognition System:* In order to determine whether or not the participant was currently smiling we employed CERT, the Computer Expression Recognition Toolbox, which provides automated real-time face detection and facial expression analysis from video [24]. The video signal used to extract this information came from two cameras, one located in each of the robot's eyes. In order to determine when a participant changed initiated or terminated a smile, we detected when the output of the smile detector had crossed a threshold (set to 0 for all participants) for at least half a second (a threshold used to make the robot's perceptions of the participant's smile less sensitive to transient noise).
